# Brief report: Aging adult utilization of an mHealth intervention for problem drinking

**DOI:** 10.3389/fpubh.2024.1462737

**Published:** 2024-10-28

**Authors:** Robyn N. M. Sedotto, Alexandra E. Edwards, Patrick L. Dulin, Diane K. King

**Affiliations:** ^1^VA Center for Integrated Healthcare, Western New York VA Healthcare System, Buffalo, NY, United States; ^2^Center for Behavioral Health Research and Services, University of Alaska Anchorage, Anchorage, AK, United States; ^3^Department of Psychology, University of Alaska Anchorage, Anchorage, AK, United States

**Keywords:** aging adult, older adult, digital health, mHealth utilization, older adult alcohol use

## Abstract

Alcohol consumption among aging adults is a growing concern due to its potential to exacerbate age-related health conditions. Developing accessible interventions for this demographic is imperative. Mobile health (mHealth) interventions offer a promising avenue, but their effectiveness and engagement among aging adults remain uncertain. This study is a secondary analysis that aimed to compare the utilization and outcomes of an mHealth intervention between aging (50+) and younger adults in a clinical trial of an mHealth intervention (Step Away) for reduced drinking. At the three-month follow-up, both age groups exhibited significant reductions in alcohol consumption and increased readiness for change. Furthermore, aging adults utilized the mHealth intervention significantly more, expressed a higher likelihood of continued use, and rated the intervention higher on the System Usability Scale (SUS). These findings suggest that mHealth interventions for alcohol-related issues can be equally effective for aging adults and that they readily engage with such tools and find them acceptable. This study underscores the potential of mHealth interventions as a viable solution for addressing alcohol-related concerns among aging adults. Further research targeting mHealth interventions tailored specifically to this demographic is warranted.

Clinical trial registration: https://clinicaltrials.gov/ct2/show/NCT04447794, Identifier [NCT04447794].

## 1 Introduction

Excessive alcohol use among aging adults is a concern, given the rising prevalence of high-risk drinking and alcohol use disorders in this demographic combined with the growing older adult population (1, 2). Aging adults are more susceptible to the effects of alcohol than their younger counterparts, due to physiological changes that occur in later life that lead to higher blood alcohol concentrations and increased intoxication levels when consuming the same amount of alcohol (3, 4). Additionally, older adults are more likely to take medications that interact with alcohol use, further increasing the risk of drinking (5).

The urgency to address this issue is underscored by the need for interventions that cater to aging adults. Mobile health (mHealth) interventions, which employ evidence-based strategies to support changes in drinking behavior, offer a promising avenue as they can increase treatment accessibility and reduce common barriers experienced to traditional treatment such as transportation, cost, and stigma (6). Among younger adults, mHealth interventions for alcohol use have been appreciated for their ability to assist in self-monitoring goals for individuals who do not have an alcohol reduction goal or those with moderation goals (7). Many aging adults report drinking above recommended guidelines but do not meet full criteria for an alcohol use disorder (8). Self-management with an mHealth intervention may be a preferred direction for these individuals. mHealth alcohol interventions also offer features that have found to be beneficial for individuals in their behavior change goals such as drink tracking, providing personalized feedback about drinking, and notifications to use the intervention (9). mHealth interventions for reducing alcohol use show promising results among younger adults (10–12), though their acceptability and efficacy among aging individuals remain underexplored (13). The application of mHealth interventions for healthy aging is an emerging area of interest (14, 15). Non-alcohol-related mHealth interventions have demonstrated success in reaching and engaging aging adults, though focus on mHealth for mental health for aging adults remains sparse (13). A better understanding of acceptability and efficacy of mHealth for reducing alcohol use for aging adults would provide additional information on a potential avenue to increase treatment accessibility and behavior change among this demographic.

The present study utilizes data from a randomized clinical trial to investigate disparities in utilization and effectiveness between aging and younger participants within an mHealth alcohol intervention however, race and ethnicity were not significantly associated with any of the designed for self-management and reduction of alcohol consumption. It focuses on creating awareness of drinking issues, setting drinking goals (abstinence or moderation), monitoring goal progress, and offering real-time assistance for alcohol-related challenges. Previous research has demonstrated significant reductions in alcohol use and related problems, along with high acceptability and usability (16, 17).

This study analyzes data from a subset of participants who were part of a larger pilot study (18) comparing the efficacy and utilization of the smartphone version of Step Away with a newly developed chatbot version. The chatbot version provided the same strategies and functions as the app but delivers them through a conversational interface on Facebook Messenger. In the larger pilot study, all participant groups experienced significant reductions in their alcohol consumption. The purpose of this study was to (1) compare mHealth utilization rates and (2) alcohol outcomes between aging adults (age 50+) and younger adults to determine if mHealth intervention is a promising direction for older individuals who want to make a change in their drinking. We hypothesized that there would be no significant differences between aging and younger adults in either utilization rates or in drinking outcomes.

## 2 Methods

### 2.1 Recruitment

This study was approved by the University of Alaska Anchorage IRB (1521800). Recruitment for the main Step Away trial was conducted through Facebook advertising from June 2020 to September 2020. A total of 155 adults participated, of which 55 were randomized to use the Step Away app, 50 to use the Step Away chatbot, and 45 to a delay condition that received no intervention. Eligibility criteria were: US residency, English proficiency, 18 years or older, had a smartphone, not in another form of alcohol treatment or using another mHealth alcohol intervention, and scored between an 8 to 24 (males under 65) or a 7 to 24 (females and males over 65) on the USAUDIT. Participants in this secondary analysis included only those randomized to an mHealth intervention [app or bot (*n* = 105)].

### 2.2 Data collection

Participants completed baseline and 12-week follow-up surveys and written informed consent through *Qualtrics* online survey platform. Participants were emailed a survey at baseline and 12-week follow-up and received a $25 Amazon e-gift card for each. Reminder emails and phone calls were made to encourage retention.

### 2.3 Measures

***Alcohol Use Disorder Identification Test – US Version (USAUDIT)***: The USAUDIT (19) is used to identify problematic drinking. A score of 8 or higher for men under 65, and 7 or higher for women or adults 65 and older (regardless of sex) has been recommended as the cutoff to detect problematic drinking (20).

***Short Inventory of Problems Revised (SIP-R)***: The 15-item SIP-R (21) is a validated measure for alcohol-related problems (22). The questions are scored on a 4-point Likert scale from 1 (never) to 4 (daily or almost daily), with higher total scores representing more alcohol-related problems.

***Timeline Followback (TLFB)***: The TLFB (23) measures alcohol consumption by participants recording the number of drinks they consumed per day for the past 30 days. The TLFB was used to calculate average drinks per day (DPD), heavy drinking days (HDD), and percentage of days abstinent (PDA). Drinking days were considered days the participant reported drinking any alcohol use.

***Readiness to Change Questionnaire (RTCQ)***: The RTCQ measures stage of change by calculating responses to 12 items and categorizing the participant into stages of change regarding changing their drinking, including precontemplation, contemplation, and action stages (24).

***Utilization***: Utilization was measured by total visits (number of times a participant used the app/bot), duration (length of time between when the app/bot was last used and first used), and active days used (number of days a participant used the app/bot). Total visits were recorded every time a participant actively interacted with the intervention (e.g., entered their daily interview, engaged in a module, reviewed their goals). This did not include if a participant opened the app and then closed it prior to interacting with it.

***System Usability Scale* (SUS)**: The 10-item SUS was used to measure user experience of either the bot or app (25). Participants were also asked to rate the likelihood that they would continue to use the intervention after the study, on a five point Likert scale (1 = not at all likely, 5 = very likely).

## 3 Analysis

Given previously reported findings that participants in both the app and bot groups changed their drinking substantially and at similar levels (18), data from the two intervention groups were combined and analyzed using SPSS 27 (*n* = 105). Participants were grouped to either an age 49 and under (i.e., “younger adult”) group, or a 50 and over (i.e., “aging adult”) group—an age cutoff commonly seen in alcohol research (26). Missing data were imputed using multiple imputation for number of drinks reported per day. Utilization data for some participants were lost due to technological glitches from the app and bot. We did not impute for utilization data. For the TLFB, some participants did not report any data (*n* = 24). We did not impute TLFB data for these participants, as all of their drinking data would have been imputed data. The change in outcome variables of AUDIT, SIP, and RTC scores from baseline to follow-up were compared via repeated measures ANOVA. Outcome variables of average drinks per drinking day, percentage of days abstinent, and heavy drinking days were non-normal in distribution, thus Wilcoxon signed-rank test was performed to determine differences between baseline and follow-up. To compare between age groups with these variables (DPD, HDD, PDA) change scores were created with the baseline scores subtracted from follow-up, such that positive numbers represent an increase and negative represent a decrease. Differences in these change scores between age groups were then compared via ANOVA. Reported intentions to continue using the app after the study, System Usability Scale scores, and usability item ratings were compared between age groups via independent *t*-tests. Utilization metrics were compared between age groups via Mann–Whitney U test due to non-normal distribution of utilization data.

## 4 Results

### 4.1 Sample

Participants (*n* = 105) identified as 1.9% Alaska Native/American Indian, 2.9% Asian, 12.4% Black, 6.7% Hispanic or Latinx, and 76.2% White. The majority identified as female (55.2%). About 30% of participants were aging adults (*n* = 31). Mean age of the younger adult group was 34.74 (SD = 7.48) and 58.45 (SD = 6.47) for the aging adult group. See Table 1 for demographics.

**Table 1:** Participant demographics.

Measure	Younger adults (Under 50; *n* = 74)	Aging adults (50+ years; *n* = 31)
Age Mean (SD)	34.74 (7.48)	58.45 (6.47)
Median (IQR)	35.00 (11)	57.00 (8)
Gender, n (%)		
Male	30 (40.5%)	17 (54.8%)
Female	44 (59.5%)	14 (45.2%)
Race/Ethnicity, n (%)		
Alaska Native/American Indian	2 (2.7%)	0 (0.0%)
Asian American	3 (4.1%)	0 (0.0%)
Black or African American	10 (13.5%)	3 (9.7%)
Hispanic or Latinx	7 (9.5%)	0 (0.0%)
White	52 (70.3%)	28 (90.3%)

### 4.2 Drinking measures

Overall, participants in the aging adult group reported significantly more drinks per drinking day (DPD) than those in the younger group at both baseline (*z* = 2.27, *p* = 0.023) and follow-up (*z* = 2.42, *p* = 0.016). Aging adults reported significantly lower readiness to change than the younger adults [*F* (103) = 4.17, *p* = 0.044]. Aging and younger adults both significantly reduced their alcohol-related problems [*F* (103) = 11.41, *p* = 0.001, η^2^ = 0.10] and increased their readiness to change [*F* (103) = 10.33, *p* = 0.002, η^2^ = 0.09] between baseline and follow-up, with moderate effect sizes. No significant interaction was found for age and time, indicating both age groups improved similarly in these drinking measures.

A Wilcoxon signed-rank test showed a significant reduction in drinking between baseline and follow-up in the overall sample, including reduction in DPD (*z* = −6.25, *p* < 0.001), reduction in HDD (*z* = −5.11, *p* < 0.001) and increase in PDA (*z* = −4.49, *p* = 0.001). When analyzing effect of age group on change in drinking, an ANOVA showed no significant differences between age groups in change in drinking scores including change in DPD [*F* (1,79) = 0.10, *p* = 0.754], HDD [*F* (1,79) = 0.06, *p* = 0.810] and PDA [*F* (1,79) = 1.27, *p* = 0.264]. Pre-intervention, follow-up, and change in drinking descriptives are shown in Table 2.

**Table 2:** Drinking measures at baseline versus follow-up.

Measure	SIP-R		AUDIT		RTCQ	
	Pre M (*SD*)	Post M (*SD*)	Pre M (*SD*)	Post M (*SD*)	Pre M (*SD*)	Post M (*SD*)
Under 50	12.19 (*8.84*)	9.00 (*7.41*)	15.59 (*3.93*)	13.16 (*6.85*)	2.31 (0*.52*)	2.55 (0*.64*)
50+	12.10 (*8.83*)	8.90 (*7.45*)	16.10 (*4.14*)	14.77 (*7.91*)	2.10 (0*.54*)	2.39 (0*.62*)
TLFB Measure	DPD		PDA		HDD	
Under 50	2.48 (*1.62*)	1.40 (*1.12*)	27.93 (*27.42*)	46.13 (*27.32*)	3.48 (*3.61*)	1.45 (*2.87*)
50+	3.00 (*1.16*)	2.03 (*1.17*)	20.57 (*20.65*)	31.14 (*28.26*)	3.96 (*4.10*)	2.12 (*3.43*)
Measure	Change in DPD		Change in PDA		Change in HDD	
Under 50	−1.08 (0.*18*)		18.24 (*28.44*)		−2.04 (*3.56*)	
50+	−0.98 (0.27)		10.57 (*28.05*)		−1.84 (*3.05*)	

For measures SIP-R, AUDIT, and RTCQ, under 50 *N* = 74 and above 50 years old *N* = 31. For DPD, PDA, and HDD, under 50 *N* = 56 and 50 above 50 years old *N* = 25. SIP-R, Short Inventory of Problems Revised. AUDIT, Alcohol Use Disorder Identification Test. RTCQ, Readiness to Change Questionnaire. DPD, Drinks per day. PDA, Percentage of days abstinent. HDD, Heavy drinking days. DPD was calculated as the average number of drinks a participant reported per day. HDD was calculated as the number of days that a participant reported 4 or more drinks for males and 3 or more for females. PDA was calculated as the percentage of days that a participant reported 0 drinks. Change variables were calculated as baseline subtracted from follow-up, where positive number indicates an increase across time points and negative indicates a decrease.

### 4.3 Utilization and acceptability

Mann–Whitney U tests were performed to compare three utilization metrics between younger adults and aging adults as the utilization variables were found to have non-normal distribution. Significant differences were found for each of the utilization metrics. Aging adults utilized the intervention almost twice as much as the younger adult group, with more total visits (*z* = 2.66, *p* = 0.008), longer duration of use (*z* = 2.10, *p* = 0.036), and more days of active use (*z =* 2.36, *p* = 0.018). Additionally, age was significantly correlated with usage in total visits (*r* = 0.45, *p* < 0.001), duration of use (*r* = 0.41, *p* < 0.001), and days of active use (*r* = 0.44, *p* < 0.001). As there was a more diverse sample in terms of race and ethnicity in the younger adult group, we compared differences in utilization rates. Due to small sample size, we combined all participants identifying as Alaska Native/American Indian, Asian, Black, or Hispanic/Latinx into one variable labeled “racial or ethnic minority” and compared their outcomes with participants identifying as White. Participants identifying as White had a significantly longer duration of use than participants identifying as an ethnic minority (*z* = 2.06, *p* = 0.040). As participants identifying as White were more heavily represented within our 50+ group, racial/ethnic identity may have impacted the duration of use difference, however, ethnicity was not significantly associated with any of the other outcome variables (Table 3).

**Table 3:** Utilization data over 90 days.

Measure	Under 50	50+		
	M (*SD*)	M (*SD*)	*z*	*p*
Total Visits	24.39 (*29.63*)	45.68 (*42.97*)	2.66**	0.008
Duration of Use	35.31 (*40.08*)	58.76 (*50.87*)	2.10*	0.036
Active Days Used	16.98 (*23.72*)	32.76 (*34.47*)	2.36*	0.018

Under 50 *N* = 62. 50+ *N* = 25. *= *p* < 0.05; **=*p* < 0.01.

Participants were asked the 10-item System Usability Scale, and item responses are shown in Table 4. Total scores were significantly lower for younger adults than aging adults *t*(98) = −2.28, *p* = 0.025, indicating aging adults in this sample rated the intervention as having greater usability than younger adults. Individual item responses between the age groups significantly differed regarding ease of use and consistency. As shown below, aging adults reported significantly lower ratings in thinking that the app or bot was cumbersome to use *t*(98) = −3.10, *p* = 0.003, and significantly lower ratings in finding the app or bot inconsistent *t*(98) = −2.08, *p* = 0.040. Aging adults also reported a higher likelihood of continuing to use the intervention after the research study compared to younger adults (*t*(98) = 2.23, *p* = 0.028), indicating greater intention of continued use (Table 4).

**Table 4:** System usability scale.

Item	Under 50	50+		
	M (*SD*)	M (*SD*)	*t*	*p*
1. I think I would use the Step Away app/chatbot frequently.	3.09 (*1.46*)	3.60 (*1.00*)	1.76	0.082
2. I found the Step Away app/chatbot unnecessarily complex.	2.71 (*1.32*)	2.17 (*1.26*)	−1.93	0.057
3. I thought the Step Away app/chatbot was easy to use.	3.59 (*1.35*)	3.97 (*1.19*)	1.34	0.183
4. I think that I would need assistance to be able to use the Step Away app/chatbot.	2.19 (*1.35*)	1.73 (*1.11*)	−1.61	0.111
5. I found the various functions in the Step Away app/chatbot well integrated.	3.33 (*1.27*)	3.53 (*1.36*)	0.72	0.471
6. I thought there was too much inconsistency in the Step Away app/chatbot.	2.76 (*1.30*)	2.20 (*1.03*)	−2.08*	0.040
7. I would imagine that most people would learn to use the Step Away app/chatbot very quickly.	3.83 (*1.09*)	4.10 (0.*99*)	1.17	0.245
8. I found the Step Away app/chatbot very cumbersome to use.	3.07 (*1.40*)	2.17 (*1.18*)	−3.10**	0.001
9. I felt very confident using the Step Away app/chatbot.	3.70 (*1.11*)	3.87 (*1.22*)	0.67	0.506
10. I needed to learn a lot of things before I could get going with the Step Away app/chatbot.	2.50 (*1.27*)	2.07 (*1.14*)	−1.61	0.111
Total System Usability Score	60.80 (*21.96*)	71.83 (*22.47*)	−2.28*	0.025

Under 50 *N* = 70. 50+ *N* = 30.

## 5 Discussion

This study sought to compare the utilization and efficacy of the mHealth alcohol intervention, Step Away, in younger adults and aging adults. All participants demonstrated substantial improvements across drinking measures from baseline to follow-up that did not differ between age groups, highlighting the intervention’s potential to facilitate behavior change with both younger and aging adults (18). Notably, aging adults not only engaged more frequently with the intervention but also expressed a higher likelihood of continued utilization after the study, reflecting a greater commitment to the program and greater acceptability of the mHealth intervention. This is a significant finding, as mHealth apps are typically developed with a focus on younger populations, and it is a common misconception that older individuals would not benefit from mHealth interventions or are resistant to using them (27). Along with this finding, aging adults also did not differ from younger adults in their reported ease of use of the app or bot. They indicated that the intervention was cumbersome significantly less than younger adults and they reported significantly higher total scores on the usability scale when asked to rate the usability of the intervention, further providing evidence against the stereotype that older adults are not able to use or do not benefit as much from mHealth interventions (27).

The heightened engagement and commitment of aging adults to the Step Away alcohol intervention also emphasizes the need for tailored features. These may include options for customizing the app’s format to accommodate vision, dexterity, and other limitations commonly associated with aging and discussed as barriers to mHealth use among older adults (15). Additionally, the integration of age-specific educational content, such as information regarding medication interactions, physiological changes, the risks of alcohol use (28), and factors predictive of AUD, like pain reliever misuse (29), could enhance the relevance and effectiveness of mHealth interventions for older populations.

It should be noted that White participants utilized the intervention for a significantly longer duration than participants of a racial or ethnic minority. It is possible the difference in diversity of race/ethnicity within the age groups may have influenced the difference in utilization shown. While total visits and active days used did not differ based on race/ethnicity, duration of use is an important utilization metric that shows sustained engagement, and may be an important utilization target for underrepresented populations. This difference highlights the importance of the current initiative to recruit ethnically diverse samples in mHealth research (30). mHealth interventions can increase access to treatment, and racial and ethnic minorities experience increased barriers to healthcare, suggesting that it is important to further investigate mechanisms to engage racial and ethnic minorities in mHealth interventions.

While the aging adults exhibited high engagement with the intervention, it is also important to note that they showed higher levels of alcohol consumption and lower readiness to change compared to their younger counterparts. This observation is consistent with prior research indicating that lower readiness to change can present a more formidable barrier to treatment among older individuals compared to younger populations (31). Nevertheless, aging participants displayed significant improvements in readiness to change from baseline to follow-up as much as younger adults did in this sample, with participants progressing from contemplation to action stages of change. This highlights the potential of mHealth interventions to foster behavior change, even in the face of initial resistance, and to potentially increase motivation to change. Overall, the findings of this study suggest that mHealth interventions to reduce alcohol use hold promise among aging adults and may serve as a valuable treatment option to address the growing need within an aging population.

### 5.1 Limitations and future research

This study has several limitations that should be acknowledged. As the bot was in early stages of development, some usage was not recorded, hence the missing data present in the usage analyses. The sample size was relatively small, with only 31 aging adults participating, as the study was not specifically designed for aging adults but drawn from a larger pilot study. A logical next step would be to conduct a study with a larger sample, including actively recruiting older individuals who are over the age of 65, and recruiting more ethnic and racial minorities. Another limitation stems from the recruitment method of convenience sampling through social media which targeted individuals who owned smartphones, potentially limiting the socioeconomic diversity and range of familiarity with technology in the sample. The participants in this study may have had greater comfort with technology than the general population. Additionally, it is possible snowball recruitment may have occurred in that those who noticed the ad could have informed others in their social circle to the study. To obtain a more representative sample of aging adults, future research could explore recruitment through healthcare providers and aging adult service organizations, which could provide technology support and outreach to a broader demographic. As this was a secondary analysis, the age group samples were not evenly distributed in ethnicity and ethnicity may have influenced the results. Results should be interpreted with this limitation in mind as these findings may not be generalizable across ethnicities. It is important in future research to recruit a more diverse sample and also stratify by race and ethnicity within age groups. Moreover, further research should delve into the specific needs and barriers faced by aging adults to develop and adapt targeted and effective mHealth interventions that consider the unique characteristics of this population.

## Data Availability

The original contributions presented in the study are included in the article/supplementary material, further inquiries can be directed to the corresponding author.
